# Supplementary material for: Divergent actions of physiological and pathological amyloid-β on synapses in live human brain slice cultures
Source: Nat Commun. 2025 Apr 30;16:3753. doi: 10.1038/s41467-025-58879-z (PMC12044016; doi:10.1038/s41467-025-58879-z)
Supplement: Supplementary file 1 — Supplementary Information [file 41467_2025_58879_MOESM1_ESM.pdf]

Supplementary tables

Supplementary Table 1: Human post-mortem case details

Diagnosis	Case ID	Edinburgh Case Number	Age (Nearest 5 years)	Sex	PMI (h)	Braak Stage
Alzheimer's disease	BBN_10591	SD003/13	85	M	76	VI
Alzheimer's disease	BBN_24526	SD055/14	80	M	65	VI
Alzheimer's disease	BBN_24688	SD058/14	95	F	61	VI
Alzheimer's disease	BBN_24527	SD056/14	80	M	74	V
Alzheimer's disease	ADRC1	-	85	F	36	VI
Alzheimer's disease	ADRC2	-	85	M	5	V
Alzheimer's disease	ADRC3	-	85	M	20	VI

BBN: UK Brain Bank Number. ADRC: Alzheimer's Disease Research Centre. PMI= Post mortem interval

Supplementary Table 2: Antibody details for array tomography

Antibody	species / isotype	product code	concentration	secondary	concentration	product code
PSD95	Guinea Pig	synaptic systems: 124-014	1:50	Donkey anti-guinea pig 488	1:50	706-545-148
OC (fibrillar-oligomeric Aβ)	Rabbit Serum	Merck: ab2286	1:200	Donkey anti-rabbit IgG 594	1:50	A21207
Synaptophysin	Goat IgG	AF555	1:50	Donkey anti-goat 647	1:50	A21447

Supplementary Table 3: Antibody details for immunofluorescence

Antibody	species / isotype	product code	concentration	secondary	concentration	product code
AT8 (p-tau Ser202/Thr205)	mouse IgG1	MN1020	1:1000	Donkey anti-mouse AF 405	1:1000	AB175658
OC (fibrillar-oligomeric Aβ)	Rabbit Serum	Merck: ab2286	1:1000	Donkey anti-rabbit AF 594	1:1000	A21207
MOAB-2 (pan-Aβ)	Mouse	Millipore: MABN254	1:1000	Goat anti-mouse AF594	1:1000	A21203
MAP2	Guinea pig	Synaptic Systems: 188004	1:1000	Goat anti-guinea pig AF 488	1:1000	A11073
NeuN	Rabbit	Invitrogen: 702022	1:500	Donkey anti-rabbit AF 488	1:1000	A21206
Iba1	Rabbit	WAKO: 019-19741	1:1000	Donkey anti-rabbit AF 488	1:1000	A21206
GFAP	Chicken	Abcam: AB4674	1:1000	Goat anti-chicken AF405	1:1000	A48260
P2YR12	Rabbit	Atlas antibodies: HPA014518	1:1000	Goat anti-rabbit AF 647	1:1000	A21244

Supplementary Table 4: Primer sequences used for RT-qPCR analysis

Oligo Name	Sequence (5' to 3')
**C1QA F**	CGAGCACCAGACGGGAAGAAAG
**C1QA R**	AGGTTCCCCCTGGTCTCCTTTA
**GAD2 F**	TGTCCAGGAAGCACCGCCATAA
**GAD2 R**	TCCTTGACGAGAATGGCAGAGC
**GAPDH F**	CCAAGGTCATCCATGACAAC
**GAPDH R**	ACAGTCTTCTGGGTGGCAGT
**GFAP F**	CTGGAGAGGAAGATTGAGTCGC
**GFAP R**	ACGTCAAGCTCCACATGGACCT
**IBA1 F**	CCCTCCAAACTGGAAGGCTTCA
**IBA1 R**	CTTTAGCTCTAGGTGAGTCTTGG
**SNAP-25 F**	CGTCGTATGCTGCAACTGGTTG
**SNAP-25 R**	GGTTCATGCCTTCTTCGACACG
**SYP F**	TCGGCTTTGTGAAGGTGCTGCA
**SYP R**	TCACTCTCGGTCTTGTTGGCAC
**SYT1 F**	GCTGACTGTTGTCATTCTGGAGG
**SYT1 R**	CTTCAGCCTCTTACCATTCTG

Supplementary Table 5: Antibodies used for western blots

Primary Antibody	Concentration	Source	Catalogue Number
Rabbit PSD95	1:500	Abcam	ab18258-100µl
Rabbit TUJ-1	1:2500	Sigma	T2200
Mouse Synaptophysin	1:500	Abcam	ab8049-500µl
Rabbit GAPDH	1:2500	Abcam	ab9485
Rabbit PGP9.5	1:1000	Abcam	ab108986-100µl
Rabbit YKL-40	1:1000	Cell Signaling Technology	47066S
Goat GFAP	1:500	Abcam	ab53554
Rabbit Cyclophilin-B	1:1000	Abcam	ab16045
Mouse APP	1:500	Merck Millipore	MAB348
Rabbit Synapsin-1	1:500	Merck Millipore	Ab1543P
Mouse SAP97	1:500	Abcam	Ab69737
Rabbit NR1	1:500	Abcam	Ab109182
Mouse GAD67	1:500	Abcam	Ab26116
Rabbit GAD2	1:500	Cell Signaling Technology	5843S
**Secondary Antibody**	**Concentration**	**Source**	**Catalogue Number**
IRDye 680RD Donkey Anti-Rabbit	1:10000	Li-Cor Biosciences	926-68073
IRDye 800CW Donkey Anti-Goat	1:10000	Li-Cor Biosciences	926-32214
IRDye 800CW Donkey Anti-Mouse	1:10000	Li-Cor Biosciences	926-32212

Supplementary Figures

a

Sections	Criteria
Macrostructure	Clear white matter and grey matter definition, ideally layer 4 visible by eye. Cortex intact, not stringy or holey.
Colour	Close to pink for grey matter, close to white for white matter. Yellow tissue not suitable.
Damage	Cauterisation from excision not used.
Tumour	No visible tumour infiltration or evidence of major angiogenesis/vascularisation.
Culture behaviour	All standard cell culture protocols followed for infection signs etc. All cultures disposed of and not used if looked to be infected.

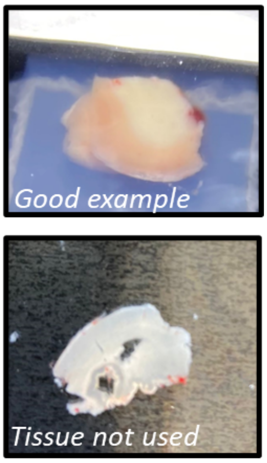

b

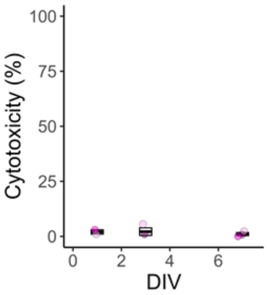

c **d**

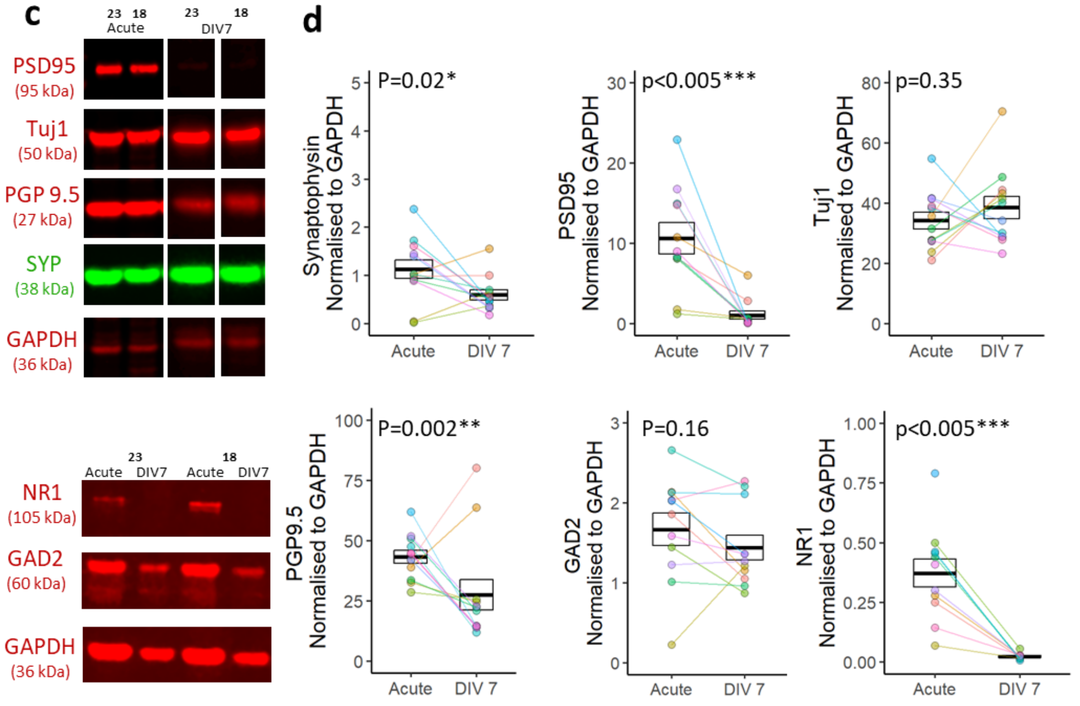

Supplementary Figure 1: Quality assessment and characterisation of human brain slice cultures. (a) *The quality control criteria used to assess whether brain slices are suitable for culture with example images from two cases (one used, one not used). (**b**) Lactate Dehydrogenase (LDH) release in medium, converted to cytotoxicity, of slice cultures over time tested at div1, 3 and 7. 100% cytotoxicity was taken as the maximal value within the*

assay from a 1% triton-x treated slice medium from the tested time points. All control samples had values less than 5% cytotoxicity. N=3 cases, with time points per div. ***c)*** *Representative western blot images from two cases for acute samples vs div 7 for PSD95, Tuj1, PGP 9.5, Synaptophysin (SYP), NR1 and GAD2 with corresponding GAPDH bands for normalisation.* ***d)*** *Quantification of acute vs div 7 samples, normalised to GAPDH. A significant reduction from acute protein expression was seen with synaptophysin (Gaussian, $\chi^2_{(1,12)}$=5.78, p=0.02*, n=12 human cases), PSD95 (Tukey, $\chi^2_{(1,11)}$=24.78, p=6.4e-07***, n=11 human cases), PGP9.5 (Tukey, $\chi^2_{(1,12)}$=9.27, p=0.002**, n=12 human cases) and NR1 (Tukey, $\chi^2_{(1,11)}$=35.87, p=2.1e-09***, n=11 human cases). No difference was seen for Tuj1 (Gaussian, $\chi^2_{(1,12)}$=0.87, p=0.35, n=12 human cases) or GAD2 (Tukey, $\chi^2_{(1,11)}$=1.96, p=0.16, n=11 human cases).* ***(b-d)*** *Data shown as box and dot plots, with box representing SEM and thick line representing the mean, with each case value joined by a colour line. Each coloured dot represents a single human case. Note: Values have been taken from different western blot assays for PSD95, Tuj1, PGP9.5 and SYP. Full western blots shown in Supplementary Source Data- Western Blots.*

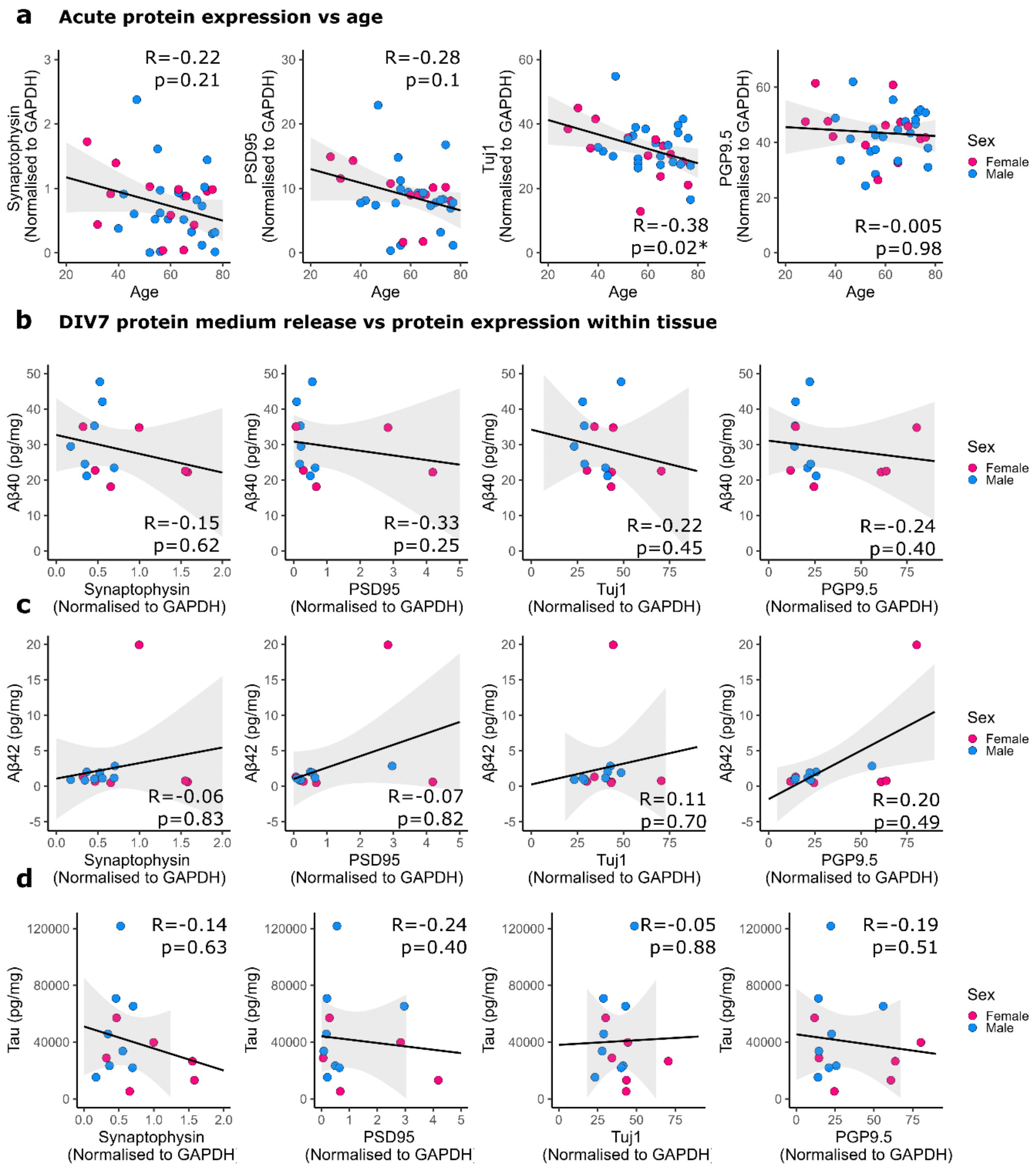

Supplementary Figure 2: Relationship between synaptic and neuronal markers and age from acute and div 7 samples. *Proteins of interest were measured by Western blot from either acute tissue (**a**) or div 7 (**b-d**) tissue and normalised to GAPDH as the housekeeping protein. Regression lines are plotted on, with shaded area representing 95% confidence intervals.* ***(a)*** *Scatter plots showing the correlations between age of patient and Synaptophysin ($r_{s(36)}$=-0.22, p=0.21), PSD95 ($r_{s(35)}$=-0.28, p=0.1)**,** Tuj1 ($r_{s(36)}$=-0.38, p=0.02*), and PGP-9.5 ($r_{s(36)}$=-0.005, p=0.98) from acute tissue. N=36 cases except for PSD95, where n=35.* ***(b)*** *Scatter plots showing the correlations between $A\beta_{1\text{-}40}$ release at div 7 and Synaptophysin ($r_{s(14)}$=-0.15, p=0.62), PSD95 ($r_{s(14)}$=-0.33, p=0.25)**,** Tuj1 ($r_{s(14)}$=-0.22, p=0.45), and PGP9.5 ($r_{s(14)}$=-0.24, p=0.40) expression at div 7.* ***(c)*** *Scatter plots showing the correlations between $A\beta_{1\text{-}42}$ release at div 7 and Synaptophysin ($r_{s(14)}$=-0.06, p=0.83), PSD95 ($r_{s(14)}$=-0.07, p=0.82)**,** Tuj1 ($r_{s(14)}$=0.11, p=0.70), and PGP9.5 ($r_{s(14)}$=0.20, p=0.49) expression at div 7.* ***(d)*** *Scatter plots showing the correlations between Tau release at div 7 and Synaptophysin ($r_{s(14)}$=-0.14, p=0.63), PSD95 ($r_{s(14)}$=-0.24, p=0.40)**,** Tuj1 ($r_{s(14)}$=-0.05, p=0.88), and PGP9.5 ($r_{s(14)}$=-0.19, p=0.51) expression at div 7.* ***(b-d)*** *N=14 cases. Pink dots are for female cases and blue dots for male cases. Statistics: Spearman's rank correlation due to non-normal distribution. Full western blots shown in Supplementary Source Data- Western Blots.*

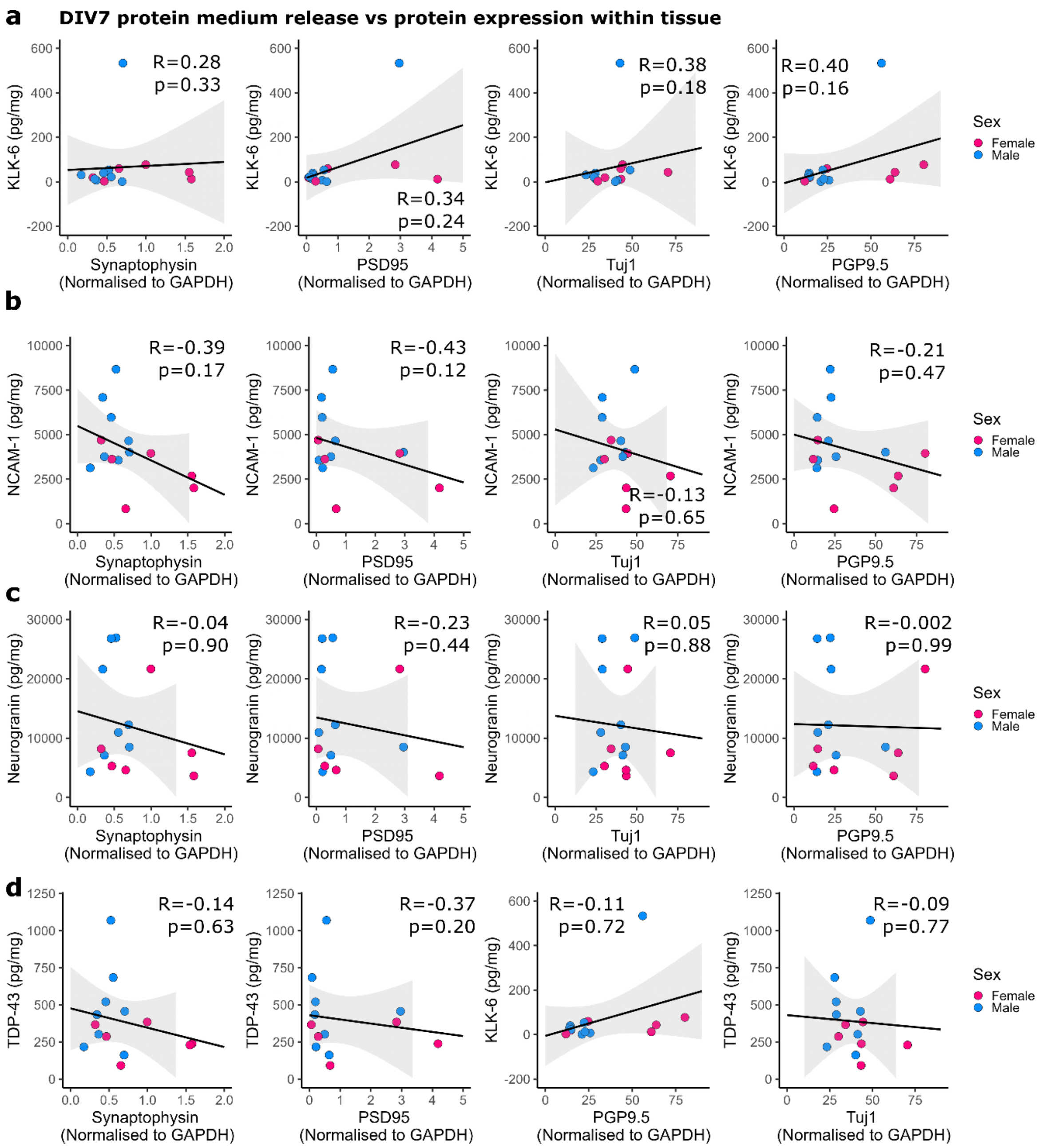

Supplementary Figure 3: Relationship between synaptic and neuronal markers from div 7 samples and biomarker release. *Proteins of interest (Synaptophysin, PSD95, Tuj1 and PGP9.5) were measured by Western blot from brain tissue at div 7 and normalised to GAPDH as the housekeeping protein. These were compared to protein release in the medium for KLK-6, NCAM-1, Neurogranin and TDP-43 at div 7. Regression lines are plotted on, with shaded area representing 95% confidence intervals.* ***(a)*** *Scatter plots showing the correlations between KLK-6 release and protein expression of Synaptophysin ($r_{s(14)}$=0.28, p=0.33), PSD95 ($r_{s(14)}$=0.34, p=0.24), Tuj1 ($r_{s(14)}$=0.38, p=0.18), and PGP9.5 ($r_{s(14)}$=0.40, p=0.16).* ***(b)*** *Scatter plots showing the correlations between NCAM-1 release and protein expression of Synaptophysin ($r_{s(14)}$=-0.39, p=0.17), PSD95 ($r_{s(14)}$=-0.43, p=0.12), Tuj1 ($r_{s(14)}$=-0.13, p=0.65), and PGP9.5 ($r_{s(14)}$=-0.21, p=0.47).* ***(c)*** *Scatter plots showing the correlations between Neurogranin release and protein expression of Synaptophysin ($r_{s(14)}$=-0.04, p=0.90), PSD95 ($r_{s(14)}$=-0.23, p=0.44), Tuj1 ($r_{s(14)}$=0.05, p=0.88), and PGP9.5 ($r_{s(14)}$=-0.002, p=0.99).* ***(d)*** *Scatter plots showing the correlations between TDP-43 release and protein expression of Synaptophysin ($r_{s(14)}$=-0.14, p=0.63), PSD95 ($r_{s(14)}$=-0.37, p=0.20), Tuj1 ($r_{s(14)}$=-0.11, p=0.72), and PGP9.5 ($r_{s(14)}$=-0.09, p=0.77). Statistics: Spearman's rank correlation due to non-normal distribution.* ***(a-d)*** *N=14 human cases. Pink dots are for female cases and blue dots for male cases. Full western blots shown in Supplementary Source Data- Western Blots.*

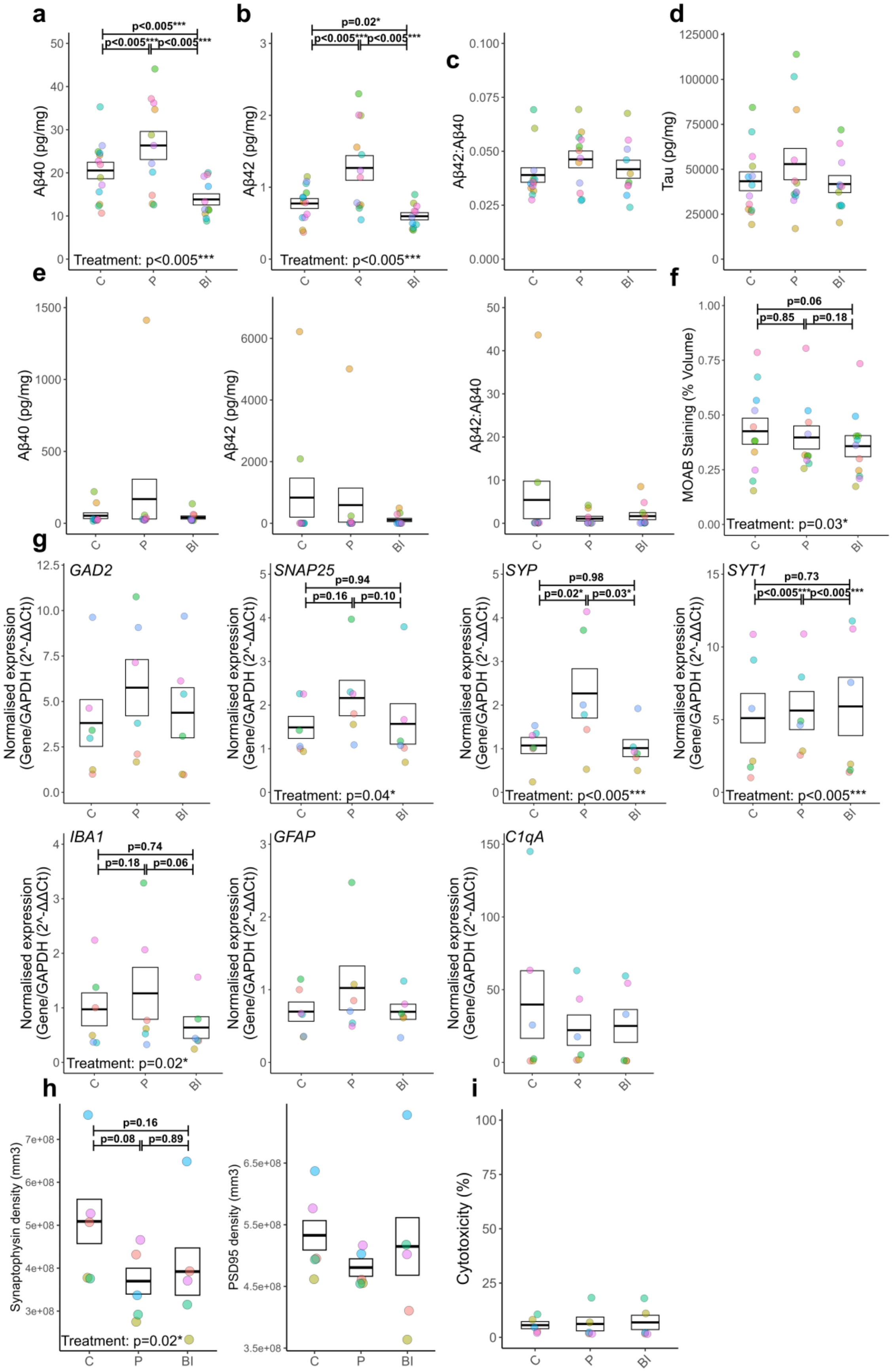

a
Aβ40 (pg/mg)
p<0.005***
p<0.005***
p<0.005***
Treatment: p<0.005***
b
Aβ42 (pg/mg)
p=0.02*
p<0.005***
p<0.005***
Treatment: p<0.005***
c
Aβ42:Aβ40
d
Tau (pg/mg)
e
Aβ40 (pg/mg)
Aβ42 (pg/mg)
Aβ42:Aβ40
f
MOAB Staining (% Volume)
p=0.06
p=0.85
p=0.18
Treatment: p=0.03*
g
GAD2
SNAP25
p=0.94
p=0.16
p=0.10
Treatment: p=0.04*
SYP
p=0.98
p=0.02*
p=0.03*
Treatment: p<0.005***
SYT1
p=0.73
p<0.005***
p<0.005***
Treatment: p<0.005***
Normalised expression (Gene/GAPDH (2^-ΔΔCt))
IBA1
p=0.74
p=0.18
p=0.06
Treatment: p=0.02*
GFAP
C1qA
h
Synaptophysin density (mm3)
p=0.16
p=0.08
p=0.89
Treatment: p=0.02*
PSD95 density (mm3)
i
Cytotoxicity (%)
C
P
BI

Supplementary Figure 4: Raw values from Figure 4. (a-i) *Box and dot plots showing responses to treatment with Phosphoramidon (P) or BACE1 (BI) inhibitor. Dots coloured based on Case ID, with each dot representing a case. Box represents standard error of the mean, with the thick line representing the mean. Statistics were performed on raw data as shown, and full statistical breakdown is in figure legend 4.* ***(a-d)*** *Responses to Phosphoramidon and BACE1 inhibitor treatment, as assessed by ELISA for $A\beta_{1-40}$* ***(a)****, $A\beta_{1-42}$* ***(b)****, $A\beta_{1-42}/A\beta_{1-40}$ ratio* ***(c)****, and tau* ***(d)****. n=36 measures from 13 cases; n=13 C, n=12 P, n=11 BI.* ***(e)*** *Protein expression for $A\beta_{1-40}$, $A\beta_{1-42}$, and $A\beta_{1-42}/A\beta_{1-40}$ within the slice, following guanidine extraction, assessed by ELISA. n=32 measures from 11 cases; n=11 C, n=10 P, n=11 BI.* ***(f)*** *MOAB-2 staining, quantified as % volume of the imaged area. n=128 measures from 10 cases; n=10 C, n=9 P, n=10 BI. Four regions across each slice were imaged and values averaged.* ***(g)*** *GAPDH normalised gene expression values (GAD2, SNAP25, SYP, SYT1, IBA1, GFAP, C1qA) in response to Phosphoramidon or BACE1 treatment from slices. n = 6 human cases.* ***(h)*** *Array tomography for synaptophysin and PSD95 densities in response to Phosphoramidon or BACE1 treatment from an additional set of cases (n = 5 human cases).* ***(i)*** *Cytotoxicity values from an additional set of cases (n = 5 human cases) to assess LDH release following treatment. No significant differences were observed (gaussian, $\chi^2_{(2,15)}$=0.68, p=0.71).*

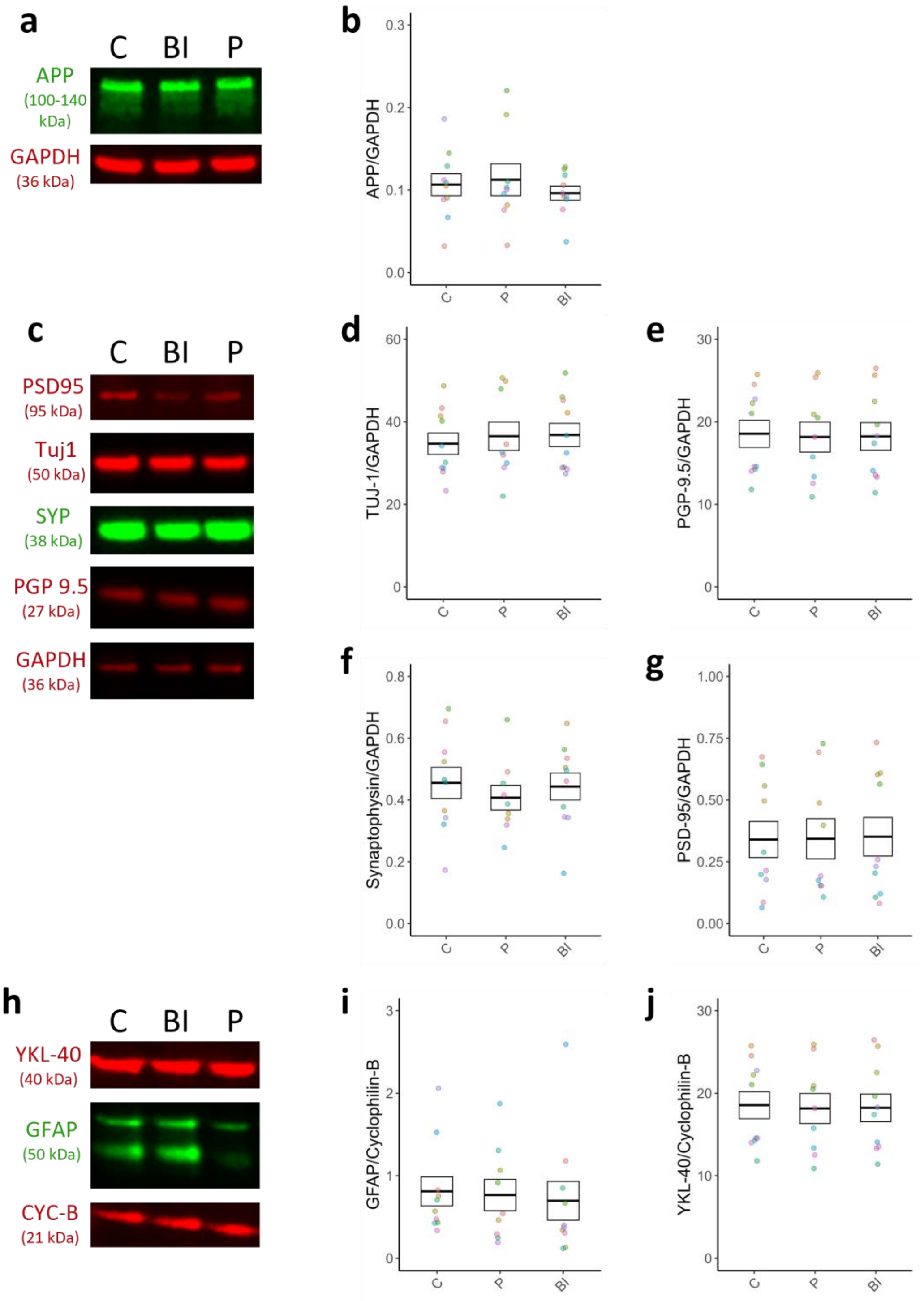

Supplementary Figure 5: Protein expression changes following Phosphoramidon or BACE1 inhibitor treatment compared to medium control. ***(a)*** *Representative Western blot and corresponding normalisation control (GAPDH) for APP, quantified in* ***(b)*** *(F=0.74, p=0.49).* ***(c)*** *Representative Western blot and corresponding normalisation control (GAPDH) for Tuj1 (F=0.71, p=0.50), PGP9.5 (F=0.13, p=0.88), SYP (F=1.05, p=0.37) and PSD95 (F=0.25, p=0.78). The quantification for this is shown in* ***(d-g)****.* ***(h)*** *Representative Western blot images of YKL-40 (F=0.13, p=0.88) and GFAP (F=0.17, p=0.84), and the corresponding normalisation control (CYC-B), quantified in* ***(i,j)****.* ***(b,d,e,f,g,i,j)*** *Statistics: LM with format var ~ Treatment + (1|Case). Box represents standard error of the mean, with the thick line representing the mean. Dots coloured based on Case ID, with each dot representing one case. C stands for control, BI stands for BACE1 inhibitor, P stands for Phosphoramidon. N=29 measures from 10 cases, 10 C, 10 BI, 9 P. Full Western blots shown in Supplementary Source Data- Western Blots.*

a

Species	Aβ +ve AD homogenate	Aβ -ve AD homogenate
Aβ1-40	28.2 pM	Below detectable limit <6 pg/ml
Aβ1-42	0.6 pM	Below detectable limit <1 pg/ml
Aβ1-42:Aβ1-40	0.02:1	N/A
Oligomeric Aβ	Below detectable limit <0.031 ng/ml	Below detectable limit <0.031 ng/ml

b

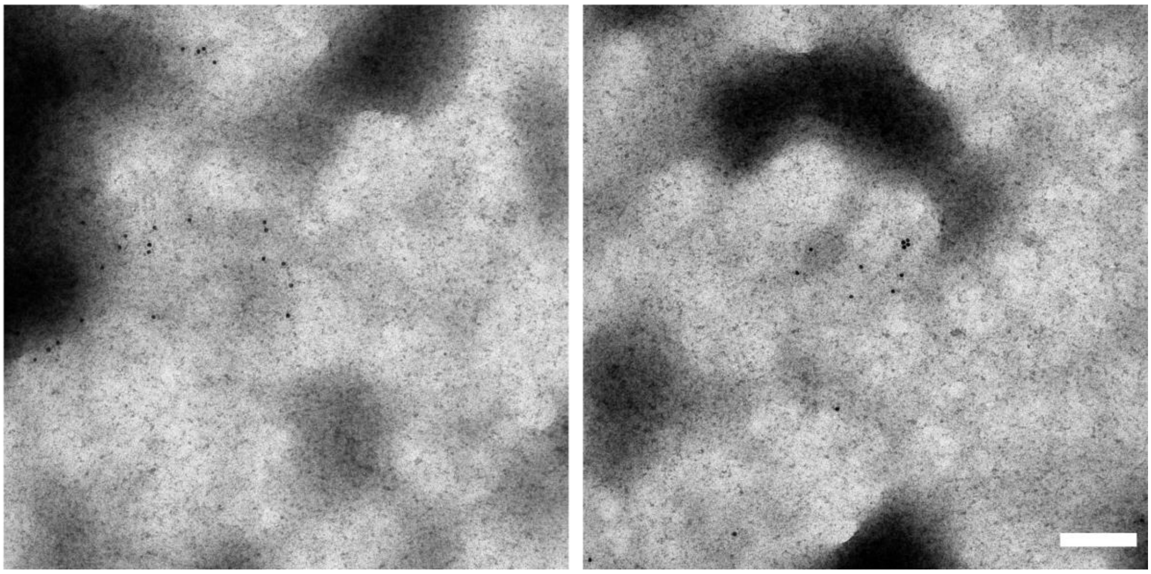

Supplementary Figure 6: Alzheimer's disease brain homogenate characterisation.
a) *Table summarising ELISA results characterising the Aβ species within the Aβ positive and negative AD brain homogenate showing successful immunodepletion of Aβ in the Aβ-ve AD homogenate and a lack of oligomeric Aβ in either preparation.*
b) *Immunogold electron microscopy with an anti-Aβ antibody reveals an absence of large fibrillar structures within the Aβ+ve AD homogenate. Positive gold staining (black dots) was seen throughout the homogenate on Aβ species with nondescript ultrastructure. Scale bar = 200 nm.*

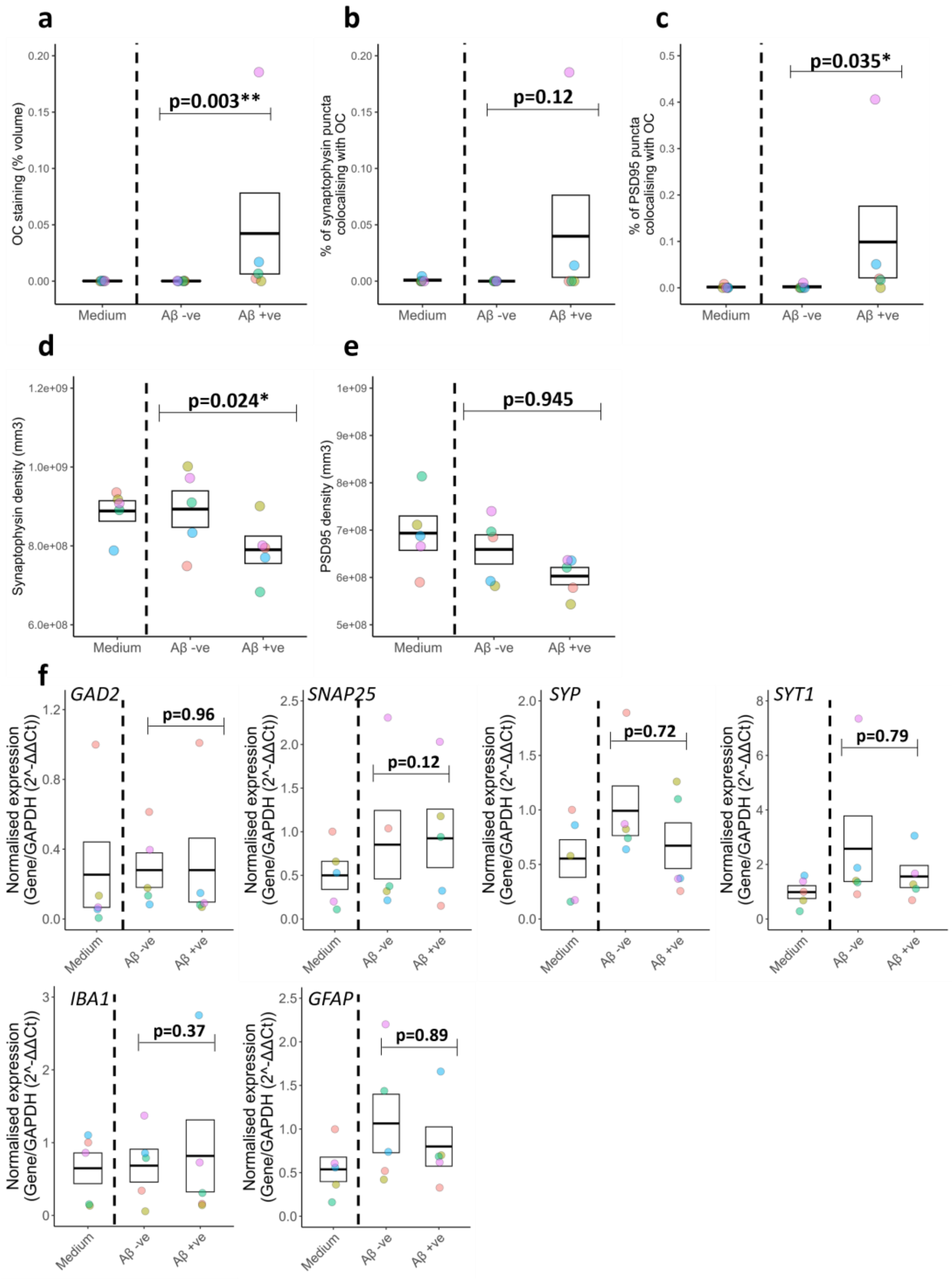

Supplementary Figure 7: Raw values from Figure 5. (a-f) *Box and dot plots showing responses to Aβ+ve and Aβ-ve soluble AD brain extract treatment and the corresponding medium control (separated by a vertical dashed line). Samples were normalised in figure 5 by subtraction of medium control to allow direct comparison of Aβ+ve and Aβ-ve AD brain extract explicitly to show the impact of Aβ treatment. Raw data is displayed here for completeness, statistical analysis was performed on data normalised to the medium only control, exactly as displayed in Figure 5. Box represents standard error and mean, with the thick line representing the mean. Colour = case. N = 5 biological replicates / human donors* ***(a-e)*** *Quantified responses to treatment with AD-brain extract compared to medium controls via array tomography. Data points refer to case means, calculated from each image stack.* ***(a)*** *Percentage volume of the 3D image stack occupied by OC.* ***(b)*** *Percentage of pre-synapses (synaptophysin) colocalising with Aβ.* ***(c)*** *Percentage of post-synapses (PSD95) colocalising with Aβ.* ***(d)*** *Synaptophysin density, expressed as mm^3.* ***(e)*** *PSD95 density, expressed as mm^3.* ***(f)*** *mRNA expression, detected by qPCR, for GAD2, SNAP25, SYP, SYT1, IBA1 and GFAP.*

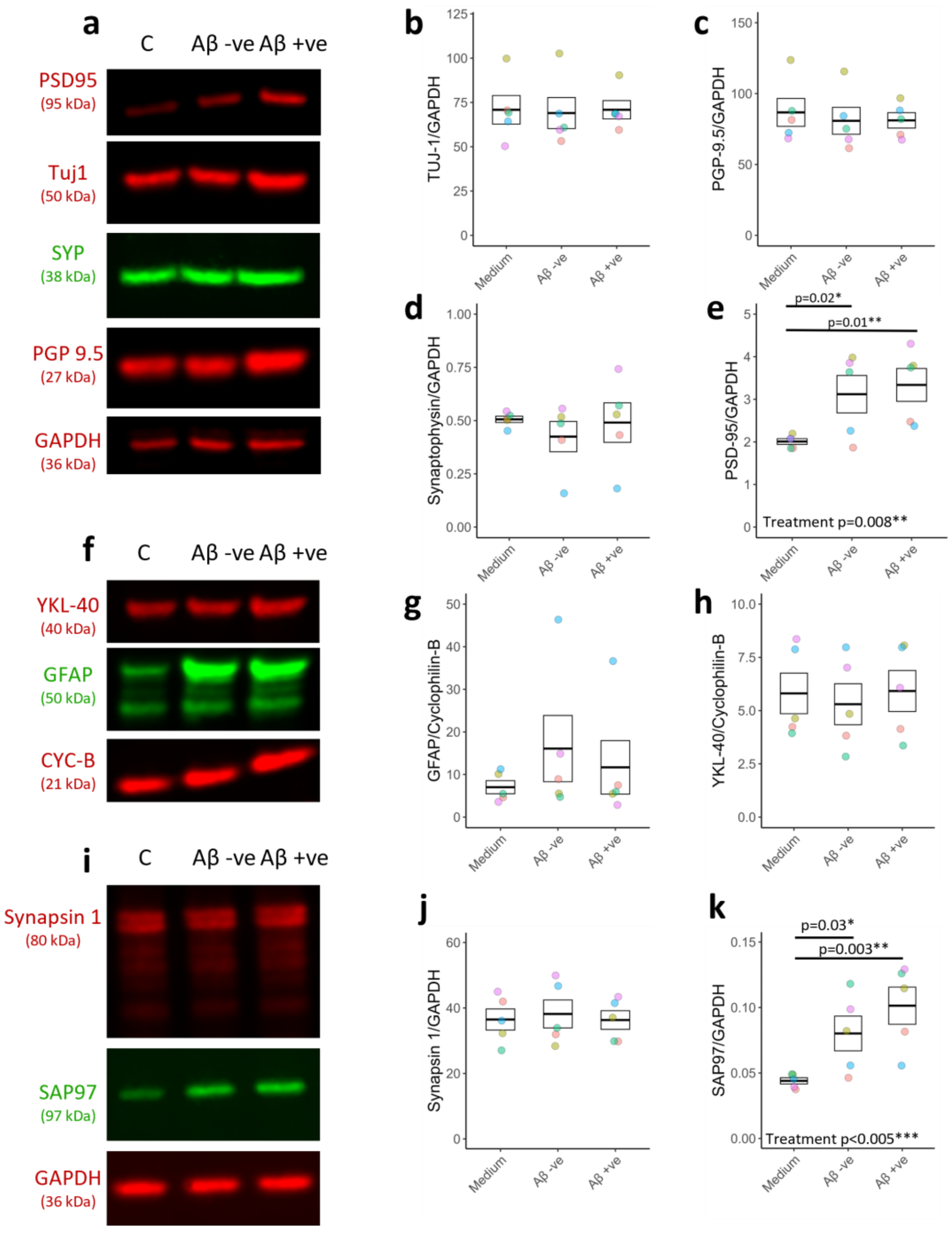

Supplementary Figure 8: Protein expression changes following Aβ+ve or Aβ-ve AD-derived brain extract treatment compared to medium control following 72 hours of treatment. ***(a)*** *Representative Western blot and corresponding normalisation control (GAPDH) for PSD95, Tuj1 (linear,* $F_{(2,15)}$*=0.11, p=0.90), SYP (linear,* $F_{(2,15)}$*=1.05, p=0.39), PGP 9.5 (linear,* $F_{(2,15)}$*=0.65, p=0.55). The quantification for this is shown in* ***(b-e)****.* ***(e)*** *PSD95 expression is significantly increased with AD-derived brain extract treatment compared to controls (linear,* $F_{(2,15)}$*=9.25, p=0.008**).* ***(f)*** *Representative Western blot images of YKL-40 (linear,* $F_{(2,15)}$*=0.45, p=0.65) and GFAP (linear,* $F_{(2,15)}$*=1.45, p=0.29), and the corresponding normalisation control (CYC-B), quantified in* ***(g,h)****.* ***(i)*** *Representative Western blot and corresponding normalisation control (GAPDH) for Synapsin 1 (linear,* $F_{(2,15)}$*=0.20, p=0.82) and SAP97. The quantification for this is shown in* ***(j,k)****.* ***(e)*** *SAP97 expression is significantly increased with AD-derived brain extract treatment compared to controls (linear,* $F_{((2,15)}$*=12.68, p=3.1e-06***).* ***(a-k)*** *C stands for control, Aβ -ve stands for Aβ immunodepleted AD-derived brain extract, Aβ +ve stands for mock immunodepleted AD-derived brain extract. Statistics: LM with format var ~ Treatment + (1|Case). Box represents standard error of the mean, with the thick line representing the mean. Dots coloured based on Case ID, with each dot representing a single case. n = 5 human cases. Full western blots shown in Supplementary Source Data- Western Blots.*

Supplementary Source Data- Western Blots

1) Blots used for Supplementary Fig 1c-d, Supp Fig 2a (Acute samples)

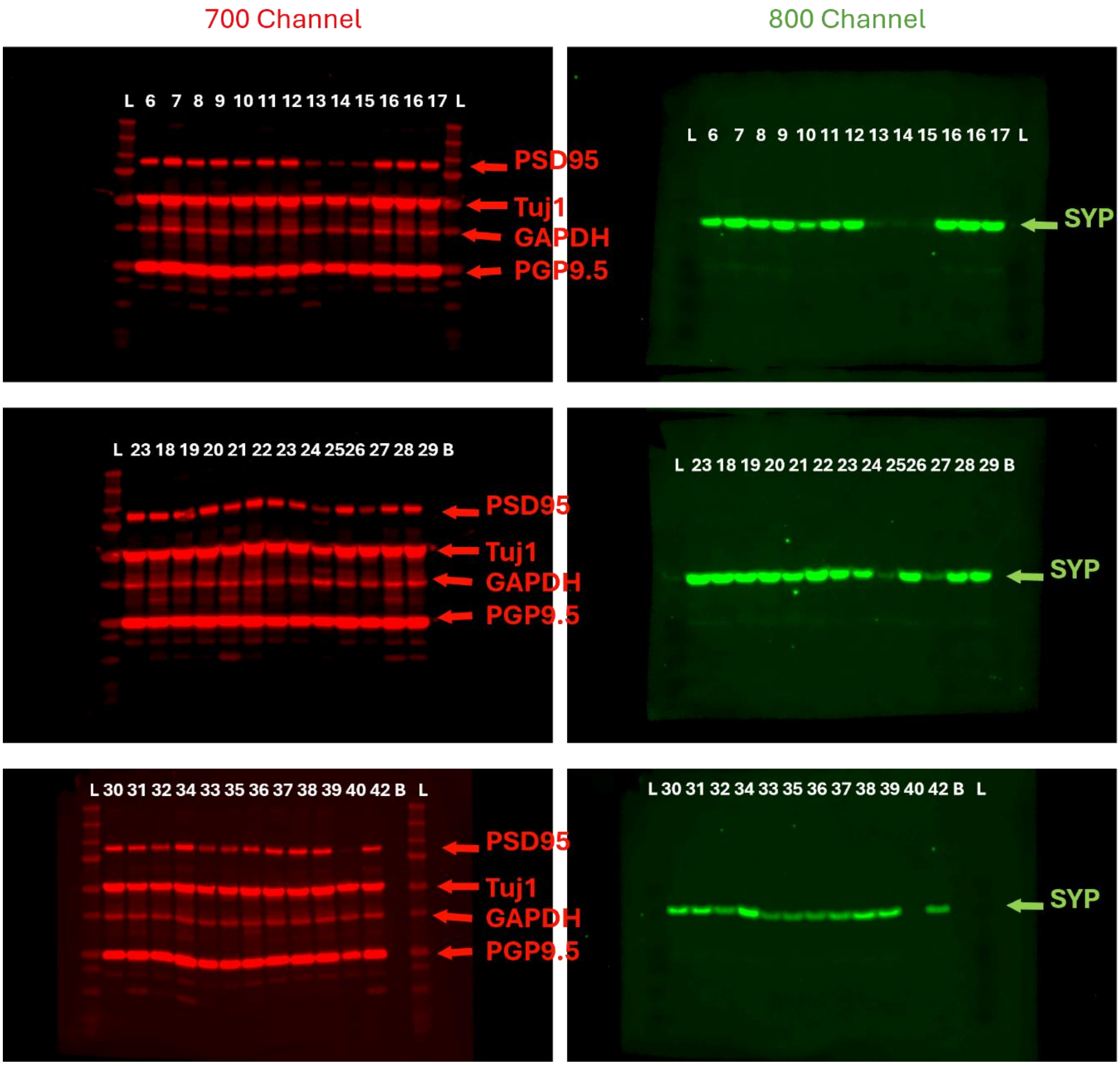

Supplementary Source Data 1: Full blots for Western blot analysis conducted in Supp. Fig. 1 c-d & 2a (Acute samples). *Blots are split into red (700) and green (800) channels. Numbers above blots represent the human case IDs, L = ladder, B = blank. Antibodies are labelled with arrows to the bands quantified in Emperia Studio. For visualisation brighter contrast images are provided. Alteration of brightness is for display purposes only and has no impact on the raw pixel values analysed in Emperia studio. Note: 2 samples were available for case 16, so both were run together (to ensure consistency between dishes) and values averaged.*

2) <u>Blots used for Supplementary Figure 1c-d (Acute and 7 div samples)</u>

700 Channel

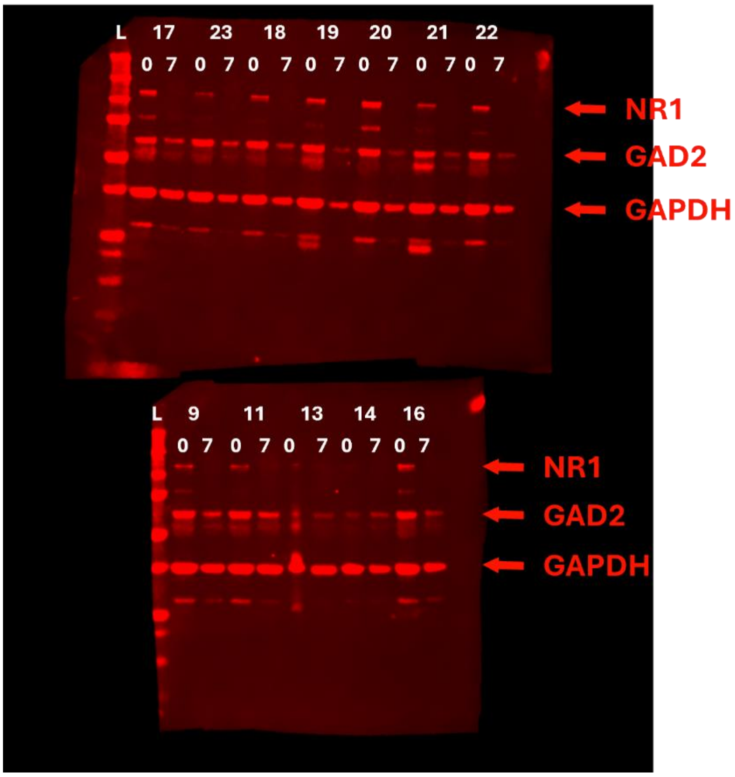

Supplementary source data 2: Full blots for Western blot analysis conducted in Supp. Fig. 1. *Blots are split into red (700) channel only. Numbers above blots represent the human case IDs, L = ladder, B = blank, 0 = Acute sample, 7 = 7 div sample. Antibodies are labelled with arrows to the bands quantified in Emperia Studio. For visualisation brighter contrast images are provided. Alteration of brightness is for display purposes only and has no impact on the raw pixel values analysed in Emperia studio.*

3) Blots used for Supplementary Figure 1c-d, 2a-d, 3a-d and 5c-g (7 div control cultures, + 7div Phosphoramidon or 7 div BACE 1 inhibitor treated cultures)

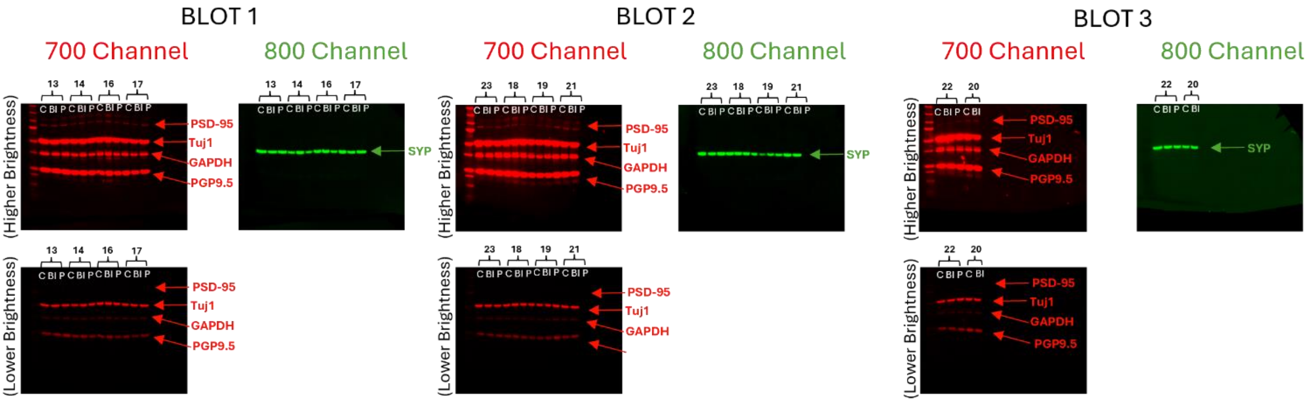

Supplementary Source Data 3: Full blots for Western blot analysis conducted in Supp. Fig. 1c-d, 2a-d, 3a-d (controls only) and supp fig 5c-g (controls, Phosphoramidon and BACE inhibitor treated). *Blots are split into red (700) and green (800) channels. Numbers above blots represent the human case IDs, with lettering in white representing the treatment condition for each case: C= Control, BI= Bace Inhibitor, P= Phosphoramidon. Antibodies are labelled with arrows to the bands quantified in Emperia Studio. For visualisation of PSD95, a brighter contrast image is provided in the 700 channel alongside the standard lower brightness image. Alteration of brightness is for display purposes only and has no impact on the raw pixel values analysed in Emperia studio.*

4) Blots used for Supplementary Figure 5a-b (7 div control, Phosphoramidon or BACE 1 inhibitor treated cultures)

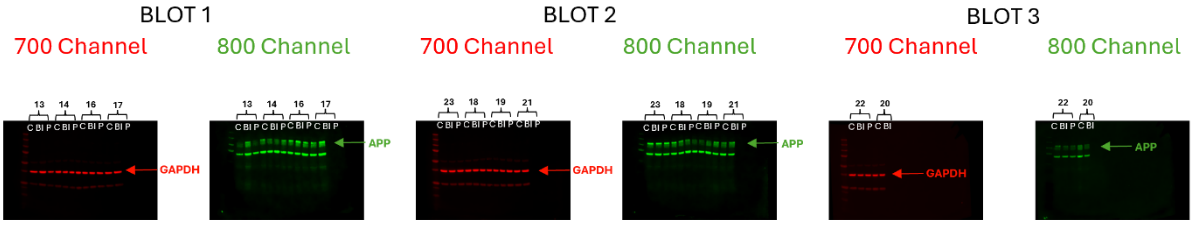

Supplementary Source Data 4: Full blots for Western blot analysis conducted in supp fig 5a-b (controls, Phosphoramidon and BACE inhibitor treated). *Blots are split into red (700) and green (800) channels. Numbers above blots represent the human case IDs, with lettering in white representing the treatment condition for each case: C= Control, BI= Bace Inhibitor, P= Phosphoramidon. Antibodies are labelled with arrows to the bands quantified in Emperia Studio. Alteration of brightness is for display purposes only and has no impact on the raw pixel values analysed in Emperia studio.*

5) Blots used for Supplementary Figure 5h-j (7 div control ,Phosphoramidon or BACE 1 inhibitor treated cultures)

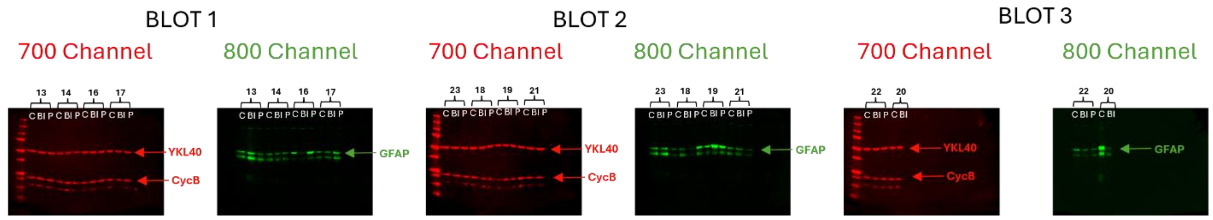

Supplementary Source Data 5: Full blots for Western blot analysis conducted in supp fig 5h-j (controls, Phosphoramidon and BACE inhibitor treated). *Blots are split into red (700) and green (800) channels. Numbers above blots represent the human case IDs, with lettering in white representing the treatment condition for each case: C= Control, BI= Bace Inhibitor, P= Phosphoramidon. Antibodies are labelled with arrows to the bands quantified in Emperia Studio. Alteration of brightness is for display purposes only and has no impact on the raw pixel values analysed in Emperia studio.*

6) Blots used for Supplementary Figure 8a-e AD-soluble extract treated cultures

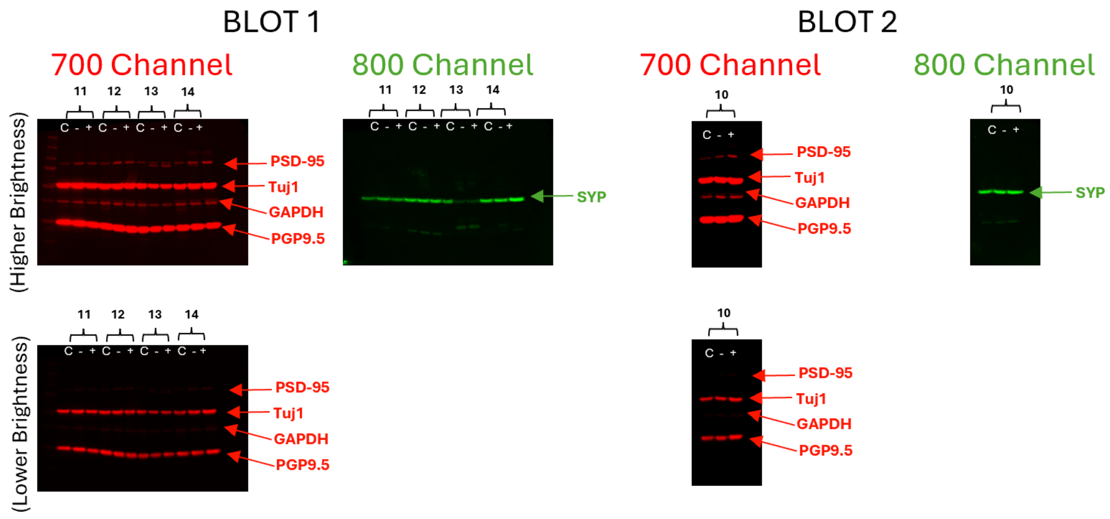

Supplementary Source Data 6: Full blots for Western blot analysis conducted in Supp. Fig. 8a-e. *Blots are split into red (700) and green (800) channels. Numbers above blots represent the human case IDs, with lettering in white representing the treatment condition for each case: C= Control/ medium only, -= Aβ – human AD brain extract, += Aβ + human AD brain extract. Antibodies are labelled with arrows to the bands quantified in Emperia Studio. For visualisation of PSD95, a brighter contrast image is provided in the 700 channel alongside the standard lower brightness image. Alteration of brightness is for display purposes only and has no impact on the raw pixel values analysed in Emperia studio.*

7) Blots used for Supplementary Figure 8f-h AD-soluble extract treated cultures

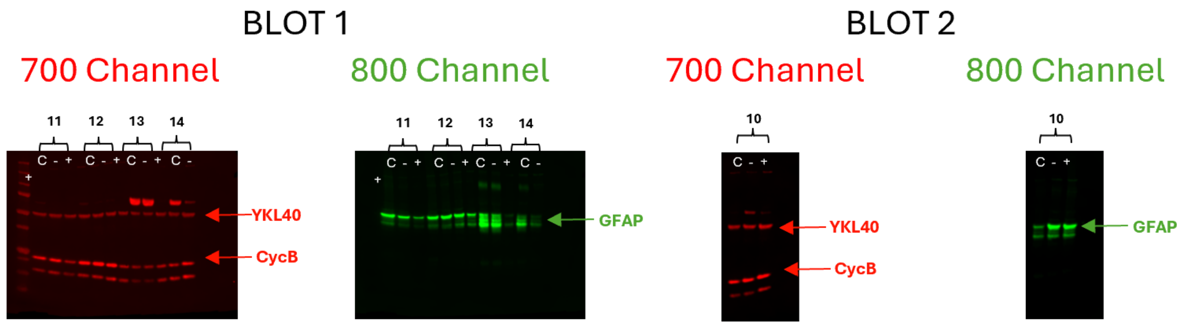

Supplementary Source Data 7: Full blots for Western blot analysis conducted in Supp. Fig. 8f-h. *Blots are split into red (700) and green (800) channels. Numbers above blots represent the human case IDs, with lettering in white representing the treatment condition for each case: C= Control/ medium only, -= Aβ – human AD brain extract, += Aβ + human AD brain extract. Antibodies are labelled with arrows to the bands quantified in Emperia Studio. Alteration of brightness is for display purposes only and has no impact on the raw pixel values analysed in Emperia studio.*

8) Blots used for Supplementary Figure 8i-k AD-soluble extract treated cultures

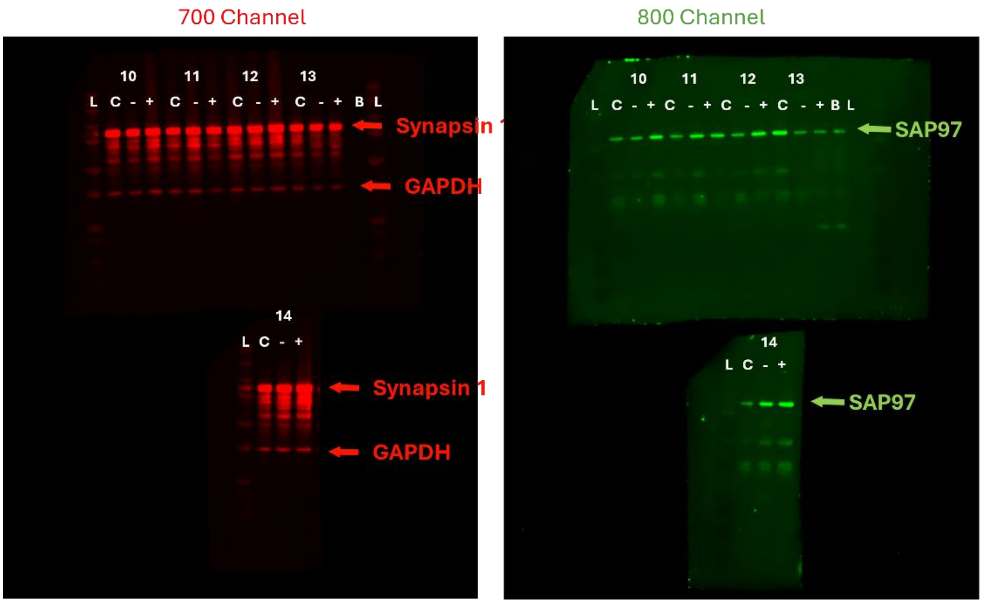

Supplementary Source Data 8: Full blots for Western blot analysis conducted in Supp. Fig. 8i-k. *Blots are split into red (700) and green (800) channels. Numbers above blots represent the human case IDs, L = ladder, B = blank, C = Medium Control, - = Aβ-ve brain extract, + = Aβ+ve brain extract. Antibodies are labelled with arrows to the bands quantified in Emperia Studio. For visualisation brighter contrast images are provided. Alteration of brightness is for display purposes only and has no impact on the raw pixel values analysed in Emperia studio.*

9) **<u>Blots used for Supplementary Figure 1d, 2 & 3. 7 div untreated cultures</u>**

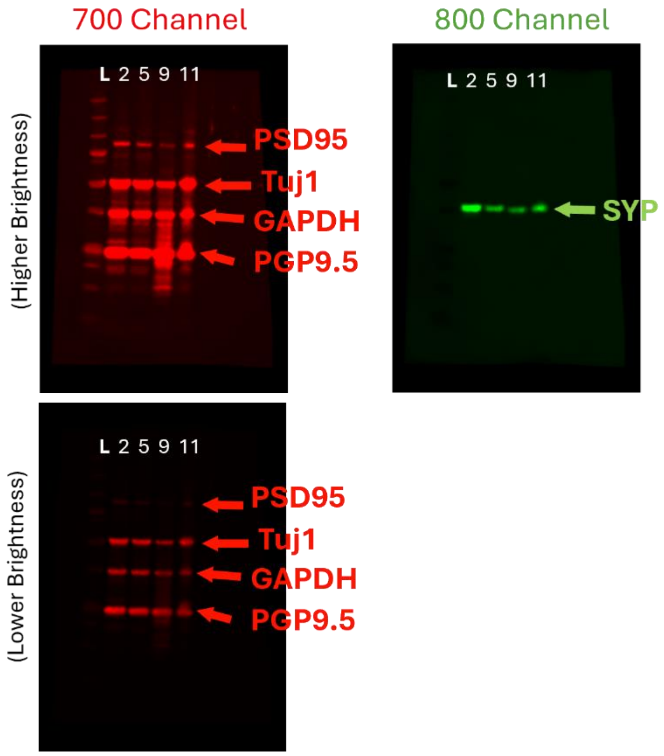

Supplementary Source Data 9: Full blots for Western blot analysis conducted in Supp. Fig. 1.d, 2 &3. *Blots are split into red (700) and green (800) channels. Numbers above blots represent the human case IDs, L = ladder. Antibodies are labelled with arrows to the bands quantified in Emperia Studio. For visualisation brighter contrast images are provided. Alteration of brightness is for display purposes only and has no impact on the raw pixel values analysed in Emperia studio. All samples here are 7 div untreated samples.*
