# Supplementary material for: Divergent actions of physiological and pathological amyloid-β on synapses in live human brain slice cultures
Source: Nat Commun. 2025 Apr 30;16:3753. doi: 10.1038/s41467-025-58879-z (PMC12044016; doi:10.1038/s41467-025-58879-z)
Supplement: Supplementary file 2 — Reporting Summary [file 41467_2025_58879_MOESM2_ESM.pdf]

nature portfolio

Corresponding author(s): Claire Durrant

Last updated by author(s): Mar 12, 2025

Reporting Summary

Nature Portfolio wishes to improve the reproducibility of the work that we publish. This form provides structure for consistency and transparency in reporting. For further information on Nature Portfolio policies, see our Editorial Policies and the Editorial Policy Checklist.

Statistics

For all statistical analyses, confirm that the following items are present in the figure legend, table legend, main text, or Methods section.

n/a	Confirmed	
[ ]	[x]	The exact sample size (*n*) for each experimental group/condition, given as a discrete number and unit of measurement
[ ]	[x]	A statement on whether measurements were taken from distinct samples or whether the same sample was measured repeatedly
[ ]	[x]	The statistical test(s) used AND whether they are one- or two-sided<br>*Only common tests should be described solely by name; describe more complex techniques in the Methods section.*
[ ]	[x]	A description of all covariates tested
[ ]	[x]	A description of any assumptions or corrections, such as tests of normality and adjustment for multiple comparisons
[ ]	[x]	A full description of the statistical parameters including central tendency (e.g. means) or other basic estimates (e.g. regression coefficient) AND variation (e.g. standard deviation) or associated estimates of uncertainty (e.g. confidence intervals)
[ ]	[x]	For null hypothesis testing, the test statistic (e.g. *F*, *t*, *r*) with confidence intervals, effect sizes, degrees of freedom and *P* value noted<br>*Give P values as exact values whenever suitable.*
[x]	[ ]	For Bayesian analysis, information on the choice of priors and Markov chain Monte Carlo settings
[ ]	[x]	For hierarchical and complex designs, identification of the appropriate level for tests and full reporting of outcomes
[ ]	[x]	Estimates of effect sizes (e.g. Cohen's *d*, Pearson's *r*), indicating how they were calculated

Our web collection on statistics for biologists contains articles on many of the points above.

Software and code

Policy information about availability of computer code

Data collection	Zeiss Zen software 3.0 (blue edition); Leica application suite X 3.5.7.23225; Licor Image Studio v 5.2; Leica Application Suite X 3.5.7.23225; pClamp 11.2; MARS; Bio-Rad CFX Maestro Software Version 2.2, xPONENT (Version 4.3, update 1), ProcartaPlex Analysis App (Luminex, ThermoFisher Connect).
Data analysis	Excel, R (version 4.3.1), MATLAB v2022a, ImageJ 2.1.0, custom scripts shared here: https://github.com/Spires-Jones-Lab.

For manuscripts utilizing custom algorithms or software that are central to the research but not yet described in published literature, software must be made available to editors and reviewers. We strongly encourage code deposition in a community repository (e.g. GitHub). See the Nature Portfolio guidelines for submitting code & software for further information.

Data

Policy information about availability of data

All manuscripts must include a data availability statement. This statement should provide the following information, where applicable:

- Accession codes, unique identifiers, or web links for publicly available datasets
- A description of any restrictions on data availability
- For clinical datasets or third party data, please ensure that the statement adheres to our policy

Data availability
1. Source data are provided with this paper.

2. Spreadsheets of analysed data are available as a supplemental data file.
3. Original western blot images are included in the supplementary data. Raw images are available from the University of Edinburgh DataVault upon reasonable request.
4. Image analysis scripts are freely available on GitHub (https://github.com/Spires-Jones-Lab).

Research involving human participants, their data, or biological material

Policy information about studies with human participants or human data. See also policy information about sex, gender (identity/presentation), and sexual orientation and race, ethnicity and racism.

Reporting on sex and gender	Sex of all donors is included in tables.
Reporting on race, ethnicity, or other socially relevant groupings	We do not have data on gender, race or ethnicity.
Population characteristics	Demographic data, including age, sex and APOE genotype are included in tables.
Recruitment	Tissue was collected from patients undergoing surgery for brain tumour resections. There is therefore a bias towards this patient subgroup. There are likely biases in self-selection for patients consenting to participation in research which we did not have control over.
Ethics oversight	Edinburgh Brain Bank ethics committee, the Academic and Clinical Central Office for Research and Development (ACCORD) and the medical research ethics committee AMREC, a joint office of the University of Edinburgh and National Health Service Lothian, Lothian NRS Bioresource, NHS Lothian Caldicott Guardian.

Note that full information on the approval of the study protocol must also be provided in the manuscript.

Field-specific reporting

Please select the one below that is the best fit for your research. If you are not sure, read the appropriate sections before making your selection.

☒ Life sciences ☐ Behavioural & social sciences ☐ Ecological, evolutionary & environmental sciences

For a reference copy of the document with all sections, see nature.com/documents/nr-reporting-summary-flat.pdf

Life sciences study design

All studies must disclose on these points even when the disclosure is negative.

Sample size	Sample sizes were determined by the availability of tissue.
Data exclusions	No data were excluded from the analysis
Replication	Our findings with BACE and Phosphoramidon are in line with data from mouse studies. We have internal replication looking at multiple patient samples for each experiment type. We have internal replication looking at multiple different synapse genes in RNA experiments, show Abeta staining with 2 different antibodies, we look at two different Abeta isoforms by ELISA. We stain for multiple neuronal or microglia markers in slices.
Randomization	Human brain slice dishes were randomly allocated to treatment groups and slices from each donor received all 3 treatments (either Control, Bace inhibitor or phosphoramidon, or control, AB- or AB+ depending on experiment)
Blinding	Experimenters were blinded where possible to treatment information during data collection and analysis.

Reporting for specific materials, systems and methods

We require information from authors about some types of materials, experimental systems and methods used in many studies. Here, indicate whether each material, system or method listed is relevant to your study. If you are not sure if a list item applies to your research, read the appropriate section before selecting a response.

Materials & experimental systems

n/a	Involved in the study
	[x] Antibodies
[x]	Eukaryotic cell lines
[x]	Palaeontology and archaeology
[x]	Animals and other organisms
[x]	Clinical data
[x]	Dual use research of concern
[x]	Plants

Methods

n/a	Involved in the study
[x]	ChIP-seq
[x]	Flow cytometry
[x]	MRI-based neuroimaging

Antibodies

Antibodies used

Primary Antibodies:

Describe the validation of each primary antibody for the species and application, noting any validation statements on the manufacturer's website, relevant citations, antibody profiles in online databases, or data provided in the manuscript.

Guinea pig anti-PSD95 Synaptic Systems Cat#124 014; RRID:AB_2619800 Lot 218
Supplier Statement: "Applications WB: 1 : 1000 up to 1 : 10000 (AP staining) gallery
IP: not tested yet
ICC: 1 : 1000 up to 1 : 3000 (see remarks) gallery
IHC: not tested yet
IHC-P: not tested yet. Immunogen Recombinant protein corresponding to PDZ-domain of mouse PSD95 (UniProt Id: Q62108). Reactivity Recombinant protein corresponding to PDZ-domain of mouse PSD95 (UniProt Id: Q62108)

Rabbit anti-OC (fibrilar oligomeric Aβ), Merck, Cat: #ab2286
References:
*Amyloid properties of the mouse egg zona pellucida. Egge, N; Muthusubramanian, A; Cornwall, GA PloS one 10 e0129907 2015
*Characterization of a Novel Mouse Model of Alzheimer's Disease--Amyloid Pathology and Unique β-Amyloid Oligomer Profile. Liu, P; Paulson, JB; Forster, CL; Shapiro, SL; Ashe, KH; Zahs, KR; PloS one 10 e0126317 2015
*Cytotoxic helix-rich oligomer formation by melittin and pancreatic polypeptide. Singh, PK; Ghosh, D; Tewari, D; Mohite, GM; Carvalho, E; Jha, NN; Jacob, RS; Sahay, S; Banerjee, R; Bera, AK; Maji, SK; PloS one 10 e0120346 2015.
*Microglia constitute a barrier that prevents neurotoxic protofibrillar Aβ42 hotspots around plaques. Condello, C; Yuan, P; Schain, A; Grutzendler, J; Nature communications 6 6176 2015.
*Forebrain microglia from wild-type but not adult 5xFAD mice prevent amyloid-β plaque formation in organotypic hippocampal slice cultures. Hellwig, S; Masuch, A; Nestel, S; Katzmarski, N; Meyer-Luehmann, M; Biber, K; Scientific reports 5 14624 2015.
*Functional amyloids in the mouse sperm acrosome. Guyonnet, B; Egge, N; Cornwall, GA; Molecular and cellular biology 34 2624-34 2014.
*Intrinsically disordered and aggregation prone regions underlie β-aggregation in S100 proteins.Carvalho, SB; Botelho, HM; Leal, SS; Cardoso, I; Fritz, G; Gomes, CM; PloS one 8 e76629 2013.
*Calcium ions promote superoxide dismutase 1 (SOD1) aggregation into non fibrillar amyloid: a link to toxic effects of calcium overload in amyotrophic lateral sclerosis (ALS); Leal, Sónia S, et al.; J. Biol. Chem., (2013) 2013.
*S100A6 amyloid fibril formation is calcium-modulated and enhances superoxide dismutase-1 (SOD1) aggregation. Botelho, Hugo M, et al. J. Biol. Chem., (2012) 2012.
*Methylene blue modulates huntingtin aggregation intermediates and is protective in Huntington's disease models. Sontag, EM; Lotz, GP; Agrawal, N; Tran, A; Aron, R; Yang, G; Necula, M; Lau, A; Finkbeiner, S; Glabe, C; Marsh, JL; Muchowski, PJ; Thompson, LM; The Journal of neuroscience : the official journal of the Society for Neuroscience 32 11109-19 2012

Goat anti-synaptophysin, R and D Systems, Cat: #AF5555, RRID: AB_2198864, Lot CCNY0222091
Supplier Statement: "Detects human Synaptophysin in direct ELISAs and Western blots...Antigen Affinity purified... Synaptophysin was detected in immersion fixed paraffin-embedded sections of human brainstem tissue using Goat Anti-Human Synaptophysin Antigen Affinity-purified Polyclonal Antibody (Catalog # AF5555) at 1.7 µg/mL overnight at 4 °C. Tissue was stained using the Anti-Goat HRP-DAB Cell & Tissue Staining Kit (brown; Catalog # CTS008) and counterstained with hematoxylin (blue). Specific staining was localized to olivary nucleus. View our protocol for Chromogenic IHC Staining of Paraffin-embedded Tissue Sections. Western blot shows lysates of human brain tissue, mouse brain tissue, and rat brain tissue. PVDF membrane was probed with 1 µg/mL of Goat Anti-Human Synaptophysin Antigen Affinity-purified Polyclonal Antibody (Catalog # AF5555) followed by HRP-conjugated Anti-Goat IgG Secondary Antibody (Catalog # HAF019). A specific band was detected for Synaptophysin at approximately 38 kDa (as indicated). This experiment was conducted under reducing conditions and using Immunoblot Buffer Group 1."

Mouse anti-AT8 (p-tau Ser202/Thr205), Invitrogen, Cat: #MN1020, RRID: AB_223647 Lot: #VL3113305
Product Specific Information: MN1020 recognizes a phosphatase-senstive epitope on PHF-Tau. No cross reactivity with normal Tau has been observed. The epitope of this antibody contains the phosphorylated Ser202 (4,5) residue (numbering according to human Tau40). MN1020 detects PHF-tau (Ser202/Thr205)a which has a predicted molecular weight of approximately 79 kDa. This product is a Low Endotoxin formulation. Purity is >95% as determined by SDS-PAGE

Mouse anti-MOAB-2 (pan-Aβ), Sigma-Aldrich, Cat: #MABN254, Lot: #3246198, NCBI accession no: NP_000475, UniProt accession no:

P05067
Specificity: This antibody recognizes amyloid-ß and not APP (amyloid precursor protein). Specifically, clone 6C3 recognizes unaggregated, oligomeric, and fibrillar forms of Aß42 and Aß40. Application: Anti-amyloid beta peptide (MOAB-2), pan Antibody, clone 6C3 is an antibody against amyloid beta peptide (MOAB-2), pan, clone 6C3 for use in western blotting, IHC (Paraffin), Immunofluorescence, IP, Dot Blot.

Guinea pig anti-MAP2, Synaptic systems, Cat: #188004, Lot: #7-52, UniProt Id: P11137-4
Specificity: Specific for MAP 2; recognizes all four isoforms. Reacts with human, rat, mouse, other species not tested yet. Applications
WB: 1 : 1000 (AP staining)
IP: not tested yet
ICC: 1 : 1000
IHC: 1 : 500
IHC-P: 1 : 200 up to 1 : 500

Rabbit anti-NeuN, Invitrogen, Cat#702022, Lot: #2694255, RRID: AB_2633050
Product Specific Information: This antibody is predicted to react with Monkey, Rat and Mouse. Recombinant rabbit monoclonal antibodies are produced using in vitro expression systems. The expression systems are developed by cloning in the specific antibody DNA sequences from immunoreactive rabbits. Then, individual clones are screened to select the best candidates for production. The advantages of using recombinant rabbit monoclonal antibodies include: better specificity and sensitivity, lot-to-lot consistency, animal origin-free formulations, and broader immunoreactivity to diverse targets due to larger rabbit immune repertoire.

Rabbit anti-Iba1, WAKO, Cat#019-19741, Lot: #PTR2404
Calcium ions are known to be one of the most important signal mediators in all cells including central nervous system (CNS) cells. Calcium ions exert their signaling activity through association with various calcium binding proteins, many of which are classified into a large protein family, the EF hand protein family.Iba1 is a 17-kDa EF hand protein that is specifically expressed in macrophages/ microglia and is upregulated during the activation of these cell. Wako has launched rabbit polyclonal antibodies were raised against a synthetic peptide corresponding to the Iba1 carboxy-terminal sequence, which was conserved among human, rat and mouse Iba1 protein sequences. Rabbit Anti Iba1 antibody is raised a synthetic peptide corresponding to C-terminus of Iba1. Purified by the antigen affinity chromatography from rabbit antisera and prepared in TBS solution. Contains no preservatives and stabilizers. Reactive with mouse and rat Iba1.

Chicken anti-GFAP, Abcam, Cat#AB4674, Lot: #GK3455458-1
Suitable for: IHC (PFA fixed), IHC-FrFl, ICC, IHC-P, WB.
IHC (PFA fixed) Use at an assay dependent concentration.
IHC-FrFl (2) 1/1000 - 1/5000. Try this antibody at about between about 1:1,000 using fluorescent secondary antibodies or 1:5,000 using peroxidase or other enzyme linked methods.
ICC 1/500 - 1/1000.
IHC-P (15) 1/200 - 1/20000. Perform heat mediated antigen retrieval with citrate buffer pH 6 before commencing with IHC staining protocol.
WB (4) 1/1000 - 1/5000. Predicted molecular weight: 50 kDa. Expect to see a band at 55kDa and another at about 48kDa, apparently a breakdown product of the 55kDa band.
Reacts with: Mouse, Rat. Immunogen: Recombinant full length protein corresponding to Human GFAP. Isotype 1 expressed in and purified from E. coli.

Rabbit anti-P2RY12, Atlas antibodies, Cat#HPA014518, Lot: #000039684.
Purinergic receptor P2Y G protein-coupled 12 (P2RY12) is encoded by the gene mapped to human chromosome 3q25.1. The encoded protein belongs to the family of P2 purinergic receptors. P2RY12 is characterized with seven transmembrane G protein coupled receptors (GPCRs) that contributes to ATP-and ADP-mediated cell migration in vitro. The protein is expressed in activated platelets and microglial cells.
All Prestige Antibodies Powered by Atlas Antibodies are developed and validated by the Human Protein Atlas (HPA) project and as a result, are supported by the most extensive characterization in the industry. The Human Protein Atlas project can be subdivided into three efforts: Human Tissue Atlas, Cancer Atlas, and Human Cell Atlas. The antibodies that have been generated in support of the Tissue and Cancer Atlas projects have been tested by immunohistochemistry against hundreds of normal and disease tissues and through the recent efforts of the Human Cell Atlas project, many have been characterized by immunofluorescence to map the human proteome not only at the tissue level but now at the subcellular level. These images and the collecation of this vast data set can be viewed on the Human Protein Atlas (HPA) site by clicking on the Image Gallery link. We also provide Prestige Antibodies® protocols and other useful information. Anti-P2RY12 antibody produced in rabbit has been used in immunohistochemistry.

Rabbit anti-PSD95, Abcam, Cat#AB18258, Lot: #1033976-1.
Rabbit polyclonal to PSD95 - Synaptic Marker
Reacts with: Mouse, Rat, Human
Predicted to work with: Zebrafish, Cynomolgus monkey, Common marmoset
Immunogen: Synthetic peptide corresponding to Mouse PSD95 aa 50-150 (internal sequence) conjugated to keyhole limpet haemocyanin (Cysteine residue).
Suitable for: ICC/IF (1-5 ug/ml), WB (1-3 ug/ml, detects a band of approximately 85 kDa (predicted molecular weight: 80 kDa), IHC-P (use at an assay-dependent concentration).
Tissue specificity: Brain.

Rabbit anti-TUJ-1, Sigma, Cat#T2200.
Clone: Polyclonal.
Tubulin β 3 class III (TUBB3) also known as β-Tubulin III (TUJ-1), is encoded by the gene mapped to human chromosome 16q24.3. TUBB3 protein expression is restricted to neurons.

α/β-Tubulin, an integral component of microtubules, is present in almost all eukaryotic cells.
α/β-Tubulin occurs mostly as soluble (approx. 100-110 kDa) heterodimeric sets of α- and β-tubulin isotypes or as polymers in assembled microtubules. β-Tubulin III (also designated β-4 chain) is found in the brain and dorsal root ganglia and appears to be localized to neurons of the central and peripheral nervous system. β-tubulin III is also found in Sertoli cells of the testis, spermatozoa tails, certain lung cells, and apparently some breast stromal cells.
Molecular Weight: Antigen: ~55 kDa.
Species Reactivity: Rat, human, mouse.
Enhanced Validation: Knockout.
Technique(s): Immunoprecipitation (IP): 10 μg using RIPA extract (250 μg) of cultured rat PC12 cells. Indirect immunofluorescence: 10-20 μg/mL using rat PC12 cells. Western blot: 0.2-0.4 μg/mL using whole extracts of mouse brain or cultured human neuroblastoma SH-SY5Y cells.
Immunogen: Synthetic peptide corresponding to amino acid residues 441-450 of human β-tubulin III (Ala446 to Ser446 substitution) with N-terminal added cysteine, conjugated to KLH. The sequence is conserved in mammals.

Mouse anti-Synaptophysin, Abcam, Cat#AB8049, Lot: #1043113-1
Mouse monoclonal [SY38] to Synaptophysin.
Reacts with: Mouse, Rat, Hamster, Cow, Human.
Immunogen: Full length native protein (purified) corresponding to Bovine Synaptophysin. Synaptophysin from presynaptic vesicles, prepared from bovine brain.
Suitable for: WB (1/500, detects a band of approximately 38 kDa (predicted molecular weights: 34 kDa)), ICC/IF (use at an assay-dependent concentration), IHC-Fr (use at an assay-dependent concentration), Flow Cyt (1/20), IHC-P (use at an assay-dependent concentration).
Tissue specificity: Characteristic of a type of small (30-80 nm) neurosecretory vesicles, including presynaptic vesicles, but also vesicles of various neuroendocrine cells of both neuronal and epithelial phenotype.

Rabbit anti-GAPDH, Abcam, Cat#AB9485, Lot: #1052911-7
Rabbit polyclonal to GAPDH - Loading Control
Suitable for: IHC-P (Use a concentration of 5 μg/ml. Perform heat mediated antigen retrieval with citrate buffer pH 6 before commencing with IHC staining protocol), WB (1/2500. Detects a band of approximately 40 kDa (predicted molecular weight: 37 kDa), ICC/IF (Use a concentration of 5 μg/ml).
Immunogen: Full length native protein (purified) corresponding to Human GAPDH.
Reacts with: Mouse, Human.
Predicted to work with: Rat, Chicken, Dog, Saccharomyces cerevisiae, Xenopus laevis, Schizosaccharomyces pombe, African green monkey.

Rabbit anti-PGP9.5, Abcam, Cat#AB108986, Lot: #1001638-2
Rabbit monoclonal [EPR4118] to PGP9.5 - Neuronal Marker
Suitable for: ICC/IF (1/500), Flow Cyt (Intra) (1/100 - 1/10000), IHC-Fr (1/250. Heat mediated antigen retrieval using sodium citrate buffer (10mM citrate pH 6.0 + 0.05% Tween-20)), WB (1/1000 - 1/10000. Detects a band of approximately 25 kDa (predicted molecular weight: 24 kDa)), IP (1/10 - 1/100), IHC-P (1/250 - 1/1000. Perform heat mediated antigen retrieval with Tris/EDTA buffer pH 9.0 before commencing with IHC staining protocol).
Reacts with: Mouse, Rat, Human.
Immunogen: Synthetic peptide. This information is proprietary to Abcam and/or its suppliers.
Positive control: WB: Fetal brain, Y79, U87-MG, SH-SY5Y, HAP1, HeLa and HEK-293T cell lysates; IHC-P: Human glioma, colon, and hepatocellular carcinoma tissue, mouse colon, mouse cerebral cortex tissue, rat Jejunum and cerebral cortex tissue; ICC/IF: Neuro-2a cells; IP: Human fetal brain lysate; Flow Cyt (intra): SH-SY5Y and Y79 cells, Neuro2a cells; IHC-Fr: Mouse cerebrum tissue.

Rabbit anti-YKL-40, Cell Signaling Technology, Cat#47066S.
Molecular Weight (Antigen): 30-40 kDa.
Suitable for: Western blotting (1:1000), immunohistochemistry (paraffin) (1:800), immunofluorescence (immunocytochemistry) (1:100).
Specificity / Sensitivity: YKL-40 (E2L1M) Rabbit mAb recognizes endogenous levels of total YKL-40 protein.
Species Reactivity: Human.
Source / Purification: Monoclonal antibody is produced by immunizing animals with a synthetic peptide corresponding to residues near the amino terminus of human YKL-40 protein.

Goat anti-GFAP, Abcam, Cat#AB53554, Lot: #GR3402854-2
Goat polyclonal to GFAP.
Suitable for: WB (Use a concentration of 0.001 - 0.1 μg/ml. Detects a band of approximately 50 kDa (predicted molecular weight: 50 kDa)).
Reacts with: Mouse, Rat, Human.
Predicted to work with: Dog.
Immunogen: Synthetic peptide corresponding to Human GFAP aa 400 to the C-terminus (C terminal) (Cysteine residue).
Positive control: WB: Human cerebellum lysates. Mouse and rat brain lysates.
Tissue specificity: Expressed in cells lacking fibronectin.

Rabbit anti-Cyclophilin-B, Abcam, Cat#AB16045, Lot: #1059088-1
Rabbit polyclonal to Cyclophilin B
Replenishment batches of our polyclonal antibody, ab16045 are tested in WB. Previous batches were additionally validated in ICC/IF and IP.
Suitable for: WB (Use a concentration of 0.5 μg/ml. Detects a band of approximately 21 kDa (predicted molecular weight: 21 kDa)),

ICC/IF (Use a concentration of 1 µg/ml), IP (Use at an assay dependent concentration).
Reacts with: Mouse, Rat, Horse, Chicken, Dog, Human
Predicted to work with: Cow, Pig, Xenopus laevis
Immunogen: Synthetic peptide corresponding to Human Cyclophilin B aa 150 to the C-terminus (C terminal) conjugated to keyhole limpet haemocyanin.

Mouse anti-APP, Merck Millipore, Cat#MAB348, Lot: #2736591
Immunogen: Purified recombinant Alzheimer precursor A4 (pre A4695) fusion protein.
Epitope: a.a. 66-81 of APP {N-terminus}.
Clone: 22C11.
Host: Mouse.
Specificity: Reacts with pre-A4. The antibody recognizes amino acids 66-81 of the N-terminus on the pre-A4 molecule (Hilbich et al., 1993). 22C11 recognizes all three isoforms of APP, immature ~110kDa, sAPP ~120kDa, and mature ~130kDa (Hoffmann et al., 2000). The antibody is known to cross react with APLP2 (Slunt, 1994).
Species Reactivity: Canine, Human, Mouse, Monkey, Pig, Rat, Fish.
Antibody Type: Monoclonal Antibody.
Application Notes: Immunofluorescence. Immunohistochemistry: 5-10 µg/mL on 4% paraformaldehyde/15% picric acid perfused and fixed rat spinal cord. 22C11 also works in paraffin embedded tissues but at lower dilutions (1:10-1:20). Western blot: reducing conditions, 10µg/ml. Optimal working dilutions and protocols must be determined by the end user.

Rabbit anti-Synapsin, Merck Millipore, Cat#AB1543P, Lot#3993513
Mouse anti-SAP97, Abcam, Cat#AB69737, Lot#GR172807-1
Rabbit anti-NR1, Abcam, Cat#AB109182, Lot#GR44063
Mouse anti-GAD67, Abcam, Cat#AB26116, Lot#GR313081-1
Rabbit anti-GAD2, Cell Signalling, Cat#5843S, Lot#1

Anti-beta Amyloid antibody [MOAB-2] (Merck, MABN254, lot: 3713390)
Quality assurance: Evaluated by Immunohistochemistry in Alzheimer's diseased human brain tissue. Immunohistochemistry Analysis: A 1:1,000 dilution of this antibody detected Amyloid Beta Peptide in human Alzheimer's diseased brain tissue.

goat anti-mouse 10nm gold-conjugated secondary antibody (Abcam, ab39619, lot: GR3379521-1). This product has been immuno-affinity purified and immuno cross-absorbed to reduce non-specific reactions. The activity of each lot is determined using a dot-spot test system as described by Moeremans et al., J. Immunol. Methods, 74, (1984), 353.

Validation

See above for validation listed below each individual antibody

Plants

Seed stocks

Report on the source of all seed stocks or other plant material used. If applicable, state the seed stock centre and catalogue number. If plant specimens were collected from the field, describe the collection location, date and sampling procedures.

Novel plant genotypes

Describe the methods by which all novel plant genotypes were produced. This includes those generated by transgenic approaches, gene editing, chemical/radiation-based mutagenesis and hybridization. For transgenic lines, describe the transformation method, the number of independent lines analyzed and the generation upon which experiments were performed. For gene-edited lines, describe the editor used, the endogenous sequence targeted for editing, the targeting guide RNA sequence (if applicable) and how the editor was applied.

Authentication

Describe any authentication procedures for each seed stock used or novel genotype generated. Describe any experiments used to assess the effect of a mutation and, where applicable, how potential secondary effects (e.g. second site T-DNA insertions, mosiacism, off-target gene editing) were examined.
